# A Case of Pernicious Anæmia

**Published:** 1889-04

**Authors:** Fred Jenner Hodges

**Affiliations:** House Gynæcologist, Cook County Hospital, Chicago


### CASE OF PERNICIOUS ANÆMIA SUCCESSFULLY TREATED WITH IRON AND ARSENIC.

BY FRED JENNER HODGES, M. D.,
HOUSE GYNÆCOLOGIST, COOK COUNTY HOSPITAL, CHICAGO.

On Jan. 5th, 1889, Augusta Johnson, a Swedish domestic, single, aged 25 years, was admitted to the Gynæcological Ward under the supposition that the marked anæmia from which she was then suffering was secondary to, or symptomatic of, some uterine affection as yet undiscovered. Her only noticable symptoms were extreme weakness and "bloodlessness." Not only was her face totally devoid of color, but her lips and the mucous membrane of her mouth were blanched and ashen.

Inquiry as to family and venereal history, habits, etc., elicited facts having only negative value. The menstrual function had been established without incident and she had always been strong and well until the spring of 1885, when she began gradually, and without evident cause, to lose strength and color, but did not emaciate in the least. These symptoms increased in severity for eight or nine months, when they began to yield to treatment. From this time her recovery was rapid and complete, she feeling as well as ever until about six months before applying at the Hospital, at which time she began again to lose color and strength, but as before did not emaciate.

Examination upon admission showed the patient to be well developed and well nourished even to the verge of obesity. The skin had a natural feel, but was waxy in appearance; the lips and buccal membrane devoid of color, presenting an ashen hue. The conjunctivæ were of pearly whiteness.

Vaginal examination showed an intact hymen, a well-formed uterus in a normal position, and a cervical canal which easily admited the sound. In other words, this examination was negative as far as determining the cause of her malady. Menses regular and with but little pain. The patient complained only of weakness following exertion, even of the slightest degree. Pulse 90, soft; temperature 99.2°; respirations about 18 or 20 a minute.

*Heart.*—Negative except that the second sound was perhaps a little shortened and accentuated. A blowing murmur was heard over the course of the great vessels of the neck, most marked on the right side. In this connection it may be said that the patient at times suffered from a pain radiating from just below the left nipple to a point beneath the right scapula. She complained also at times of a slight breathlessness upon going up even a few steps, but there was not the slightest œdema of the extremities nor has she ever suffered from rheumatism.

*Lungs.*—Examination entirely negative.

*Spleen.*—This organ was slightly enlarged.

*Kidneys.*—Urine acid, sp. gr. 1025, and contained no albumin, casts, or sugar. Was considerable in quantity and pale in color.

*Stomach*, *Pancreas*, etc.—There was almost complete anorexia, yet food once taken did not cause distress. There was a particular aversion to fatty foods, yet an examination of the fæces showed no failure of the pancreatic function. Bowels had as a rule been sluggish.

*Ophthalmoscopic examination.* — Each retina showed numerous yellowish spots having bluish-gray areolæ. Their average size was that of the macula, and they were most numerous in its locality. The disks were somewhat swollen and quite anæmic.

Microscopical examination of the blood showed (a) red corpuscles of the usual size and form; (b) white corpuscles in the proportion of 1 to about 85 or 100 red; (c) numerous irregular (rod- and kidney-shaped, globular and tailed) red corpuscles; (d) numerous highly refractive coccus-like bodies appearing much as would globules of oil; (e) irregular masses of a brownish-red amorphous material.

*Diagnosis.*—Idiopathic or pernicious anæmia, with an actual leucocytosis.

Desiring to test the efficacy of iron and arsenic in this class of cases the patient was put upon the following:

℞

Massa ferri carb..........ʒij

Liq. pat. arsen............sʒiss

Syrup. limonis............s℥iij

Aquæ.............g.s. ad s℥iv

Rub the mass thoroughly with the syrup, add the Fowler's Solution, and direct:

Teaspoonful four times a day. She also received at night a pill containing aloes, nux vomica, piperine, etc.

Two weeks afterward I find this entry on the history: "Lips begin to show color, patient says she feels better and has some appetite"; and one month from the time of her entrance she was discharged sufficiently recovered to enable her to engage in light work for the entire day without feeling the worse for it. An examination of the blood at this time fails to show further abnormalities than a slight excess (proportionately) of white corpuscles.

COOK COUNTY HOSPITAL.

